# The Girl Who Wants to Get Rid of Her Left Leg—Body Identity Dysphoria

**DOI:** 10.3390/healthcare11131901

**Published:** 2023-06-30

**Authors:** Erich Kasten

**Affiliations:** Department of Psychology, Faculty of Human Sciences, Medical School Hamburg, 20457 Hamburg, Germany; erikasten@aol.com

**Keywords:** body identity dysphoria, body integrity identity disorder, apotemnophilia, amputee identity disorder, xenophilia

## Abstract

Introduction: One of the strangest kinds of misperceptions of the body is “Body Integrity Dysphoria” (BID), formerly named as “Body Identity Integrity Disorder” (BIID). The affected people have the feeling that a limb is not part of their body. They can feel it, they can use it, they can move it, but they cannot get along with the fact that it is a part of their own body. Most feel the need for an amputation of a leg, others of both legs, some want a palsy and use a wheelchair. Still discussed is whether other disablements such as blindness, dumbness, deafness or a desire to have an incontinency can be included in this diagnosis. This review discusses parallels and differences to transgender/trans identity, body dysmorphic disorder, alien limb syndrome, hemineglect, and self-induced amputations in schizophrenic patients. The cause for the need to be disabled is still unknown; the review gives an overview about psychological and neurological theories of explanation and what kind of therapy may help. Methods: This is a narrative review of about 20 years of research about Body Integrity Dysphoria by the author. Results: Overall, no psychopathological deviations were found, none of the affected persons examined by us were actually delusional or schizophrenic, which underlines that there is a neurological malfunction in the brain that has existed since birth. However, psychological mechanisms intensify the symptoms. There are clear parallels to other forms of interference between the external body and mental body representation. Different types of therapies have been able to provide help to better deal with BID, but there has been little to bring about a real cure. In contrast, BID-affected persons who achieved amputation (or other desired forms of disability) were satisfied and able to return to work. Conclusions: BID remains an enigmatic disorder. We have learned a lot over the past 20 years that the mental and physical bodies do not have to match.

## 1. Introduction and Background

### 1.1. Example

This article is a narrative review of about 20 years of scientific research of the author about a strange phenomenon: People who want to get rid of parts of their body or to suffer from paralysis or blindness. To start with an example, in 2018, a new patient came to see me. She was 28 years old. Although she was slim, small and attractive, she worked as a craftsman in a demanding job. She lived with her boyfriend, and she was mostly cheerful, sociable and open-minded. In addition, she wanted to get rid of her left leg. She felt that this leg was not part of her body. At that time, I did an examination with various psychological tests, but I could not find any psychopathological disorders. In my mind, she was perfectly normal in every way. In regard to her wish for an amputation, her boyfriend was helpless, believing it was just a “whim” that would go away. I had little contact with her after that. In 2022, she surprised me that she had broken up with her boyfriend and was in a clinic abroad, where a surgeon had amputated her left leg from mid-thigh. Although she subsequently developed an infection in her leg and had to have antibiotics and reoperations, and although she had given up her job and her boyfriend, she was happier than ever and highly satisfied with her leg stump.

### 1.2. Feeling the Need for an Amputation

The feeling of a need to have a body part amputated was initially described as “apotemnophilia”, later as “amputee identity disorder”, then as “body integrity identity disorder” (BIID), “xenophila”, and now it is named “body integrity dysphoria” (BID).

BID sufferers are not concerned with looking more beautiful after an amputation, they suffer considerably, because their intact external body does not correspond to their mental representation of a disabled body.

At first glance, this is absolutely incomprehensible to the average citizen: how can one be so crazy as to wish the amputation of a healthy part of the body? An article in a newspaper reveals that a 43-year-old woman demanded EUR 100,000 in compensation for pain and suffering because her leg had to be amputated due to a doctor’s mistake. Most patients suffer from losing a body part or being paralyzed in a wheelchair. What makes a person feel a need for such an irreparable condition?

### 1.3. What Is Body Integrity Dysphoria?

To tell the sad thing first, the answer to the question of what Body Integrity Dysphoria (BID) actually is remains nebulous. “*The soul feels it belongs to a body with only one leg or one arm. The body does not correspond to this inner reality*”, one person described the feeling. In the 1970s, the phenomenon was called “Apotemnophilia” (i.e., “love of cutting off”, [1]), later emerged the name “Amputee Identity Disorder”. However, it quickly became clear that this strange need was not just about amputations, but also about paralysis, especially paraplegia. The American psychiatrist Michael First [2] created the broader term “Body Integrity Identity Disorder” (BIID), under which most scientific publications can still be found today. Later, “Xenophilia” (from “xeno” = foreign) was added, but it is rarely used. Those affected sometimes call themselves “Wannabes” (from want-to-be). In 2004, the American psychiatrist Michael First [2] summarized as the most important criteria that the urge to change the body existed since childhood/adolescence and that those affected did not appear delusional in any way.

Whether other handicaps such as, for example, the need to be blind, deaf, toothless, incontinent, mute or stuttering, really belong to BID is currently the basis for escalating discussions among experts. However, our most extensive study to date, carried out by Maria Garbos on 213 affected people [3], shows that the need for amputation and paralysis accounts for the largest part at around 85% (see Table 1).

In addition to the need for amputation and palsy, in our studies, we also looked at the neighboring areas of disablement such as, for example, incontinency, deafness, dumbness or to become toothless; the need for blindness will be discussed later in this review. In his work on the topic “The desire to be edentulous”, Sebastian Heim [4], from our working group, quoted some statements about the motivation from those affected with a desire to get rid of their teeth: “*For me, removing the teeth is the necessity of wearing dentures and wearing dentures has always been an absolute wish* […] *I love this feeling of plastic and the super-smooth denture palate* […] *For me it was and is the absolute fulfillment to be toothless and to be able to wear these great dentures; I should have done it much, much earlier*”, said one of the people interviewed. A second responded similarly: *“I wish nothing more and more in the world than finally removing the remaining teeth, crowns and bridges and replacing them with removable dentures* […] *I want snow-white and perfectly straight teeth and pink plastic gums, a big, thick palate!”* The statements are certainly in line with what is typical of BID, but on the other hand, they do not explain the real “why”.

### 1.4. BID and the International Classification of Diseases

In many countries, diagnoses are made using the “International Classification of Diseases”. Since January 2022, the new version ICD-11 has been valid, which includes BID. Here, it says in the section “Disorders of bodily distress or bodily experiences”:
6C21 Body integrity dysphoria. Body integrity dysphoria is characterized by an intense and persistent desire to become physically disabled in a significant way (e.g., major limb amputee, paraplegic, blind), with onset by early adolescence accompanied by persistent discomfort, or intense feelings of inappropriateness concerning current non-disabled body configuration. The desire to become physically disabled results in harmful consequences, as manifested by either the preoccupation with the desire (including time spent pretending to be disabled) significantly interfering with productivity, with leisure activities, or with social functioning (e.g., person is unwilling to have a close relationship because it would make it difficult to pretend) or by attempts to actually become disabled having resulted in the person putting his or her health or life in significant jeopardy.

For people suffering from BID, the inclusion in the ICD-11 was one of the greatest achievements in recent years, and there is great hope that if BID is a recognized diagnosis, health insurance companies have to provide and pay for treatment. However, currently, the cost bearers are demanding scientific effectiveness studies according to the gold standard of research (i.e., randomized, blinded control group studies) which clearly demonstrate that an amputation actually, and even in the long term, cures the condition. In this regard is an interesting question regarding how to have a placebo control group for amputations. However, until now there has been only one published study by Sarah Noll et al. (2014) that has devoted itself to this topic, and it is based on a survey of 19 BID sufferers who have achieved their goal [5]. At first glance, this number seems small; however, since BID is an extremely rare disorder, this study is still valuable. The data from this study will be presented later. The data of another study of Buket Saricicek [6] are presented in this special issue about “misperceptions of the body” for the first time.

Until now, the succinct problem is that the amputation of a healthy part of the body is, in principle, in most countries illegal and may even be a criminal offence. At least 17% of patients in the abovementioned publication by M. First [2] had actually been able to achieve their desired state. A scandal broke out in 1999 when Scottish doctor Robert Smith performed amputations on two BID sufferers, but the press found out about it, and further operations of this kind were then forbidden by the hospital management. In order to initiate legal medical surgery of BID in Germany (and possibly the rest of the world), one would have to scientifically provide evidence that a currently illegal amputation of a medically healthy leg brings the most effective benefit. Then, the cat bites its tail: How can you prove that a method that is illegal and should not be done at all brings the best benefit? 

However, most surgeons are currently reluctant to cut off a healthy leg, because they are afraid of negative media hype and, in the worst case, fear losing their license to practice medicine. Insurance companies are less concerned about the cost of the surgery itself, but much more about lengthy follow-up costs for special prostheses, wheelchairs, physiotherapy, treatment of phantom and residual limb pain and retraining in other occupations, which is often necessary. Therefore, there is not much enthusiasm on the part of physicians or health insurance companies to quickly introduce a legal amputation. 

In addition, there is no purely medical indication for the amputation, since the leg is organically healthy. However, there is absolutely a psychological indication, because the mental stress can be excruciating. The level of suffering is evident in examples such as that of the Australian citizen David Openshaw, who stuck his leg in dry ice for 6 h, forcing doctors to perform an amputation. Other sufferers have used far more drastic methods, which shows how badly these people suffer from having a limb that they feel is not part of themselves.

### 1.5. Public Relations

Even expert opinion on BID is changing only hesitantly. According to a press release, psychology professor Winfried Rief considered the amputation to be “*unacceptable*” in 2000, and his colleague Ulrich Stangier named Dr. Smith’s operation in Scotland as “*dubious”.* In 2009, together with Dorothee Neff from the Royal Holloway University in London, we published a study in which we presented the typical description of a BID patient to German and English specialists and asked them to make a diagnosis [7]. Only a fraction correctly identified the symptoms; almost 45% of the English and 25% of the German experts were in favour of better accommodating such people as inpatients in a psychiatric clinic. Well over 60% of the professionals from both countries categorically refused to assist this patient to achieve the desired amputation. Over the past 15 years, we have worked extremely hard, with countless articles in the professional world as well as in the tabloid press, radio and TV shows, to achieve better education about Body Integrity Dysphoria among both professionals and the general public. Around 10 years after the German–English publication, Sabur Safi went to a busy shopping street in Hamburg (Germany), read the typical BID description to 133 passersby and asked for an assessment [8]. After all, around 84% of these people on the street classified BID as a “mental illness”. On the other hand, almost half of the passersby said that everyone has the right to freely make decisions about their own body, even if they wish to be mutilated, and 42% even stated that people affected by BID should be supported.

## 2. Studies

### 2.1. Types and Strengths of Symptoms of BID

From a lapidary cold to cancer, no illness is like the other. This also applies to BID. Apparently, there are people concerned who suffer so badly that they put their leg on a rail and wait for the next train; on the other hand, there are BID people who can handle the pressure lifelong. In 2013, Mona Fischer published a first version of a questionnaire to record the severity of the suffering associated with BID [9]. Here, it was already shown that the strength of the need for amputation resembled a normal distribution. The questionnaire has now been significantly revised by Maria Garbos [3], after criticism was levelled that the older version actually only talked about amputation. The current questionnaire by Maria Garbos (2022) from our group covers all types of disabilities and asks about the effects on different areas of life.

**Figure 1:**
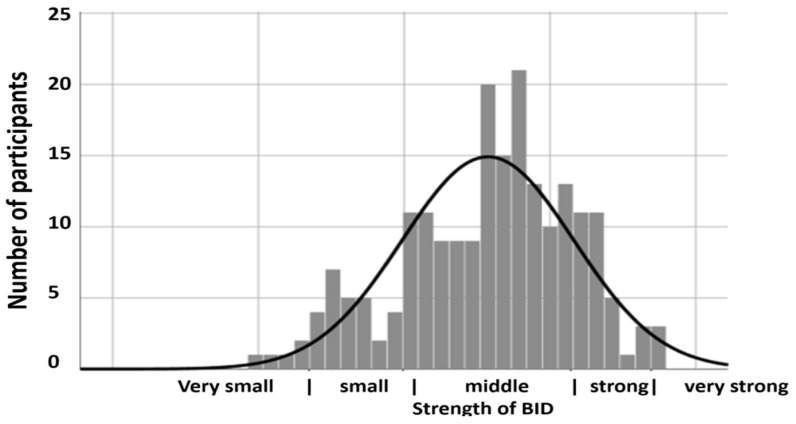
Distribution of the severity of BID in Maria Garbos’ questionnaire.

Figure 1 shows that there is a small number of those affected with mild forms, the middle field is most occupied and only a comparatively small number suffer from very severe forms [3].

**Figure 2:**
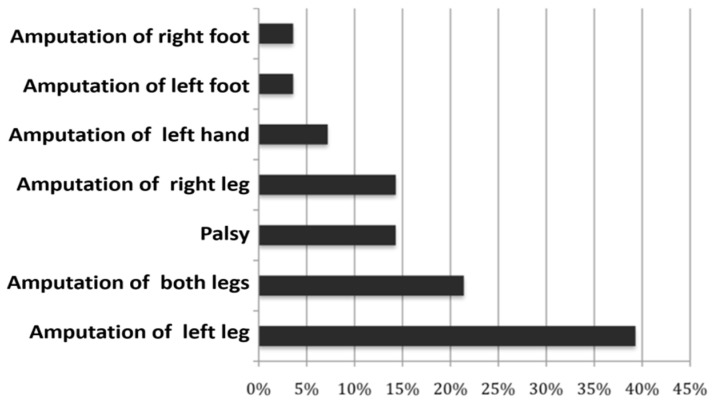
Frequencies of the need for amputation and paralysis in the study by Kasten and Spithaler [10].

BID differs not only in the severity of the need, but much more in the type of disability. Fredrike Spithaler from our group, with the help of Rachel Esterhazy, had already conducted a study on this in 2009, which, however, was primarily aimed at amputation and paralysis [10]. What is striking in Figure 2 is that the left leg is primarily the target object. Neurobiological causes were discussed for this, but those affected themselves sometimes admit that they need their right leg to drive a car and that it is therefore wiser to keep it.

**Figure 3:**
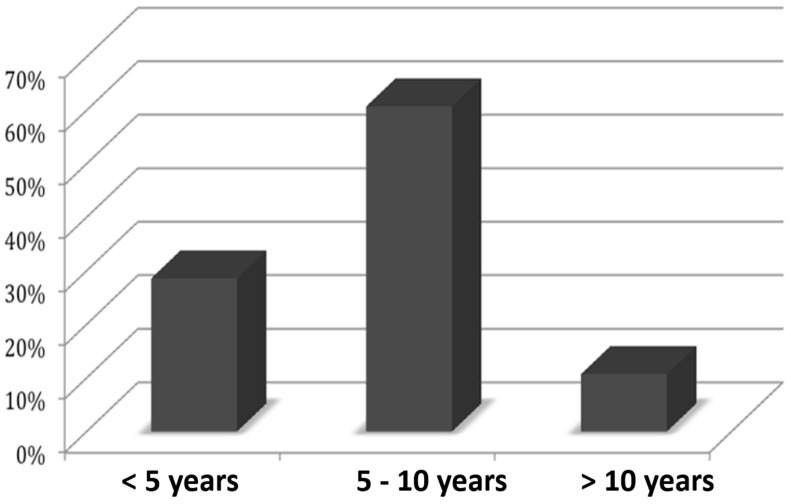
Beginning of the awareness that something is wrong with one’s own body in the study by Kasten and Spithaler [10].

The authors of this study also asked at what age it was first noticed that the internal body image did not match the external one. Figure 3 shows that the peak of the frequency distribution is between the ages of 6 and 10.

### 2.2. Eroticism, Sexual Orientation and BID

**Figure 4:**
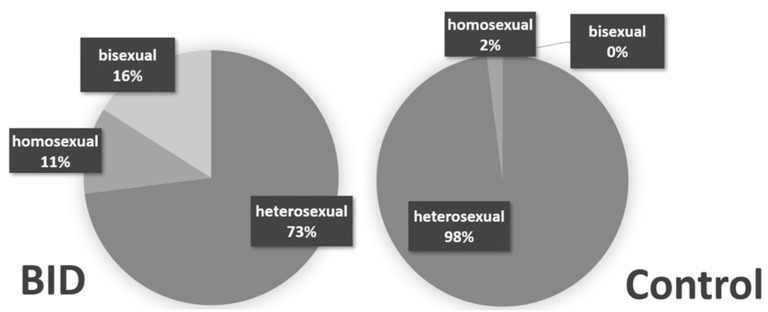
Distribution of the sexual orientation of a BID group (**left**: 12 women, 31 men, 1 diverse) and a control group of unaffected people (**right**: 11 women, 33 men, 0 diverse) in Diana Becker’s study [11].

Another point, which probably causes headaches not only for the experts, is a strange gender influence as well as erotic undertones of BID. In the primeval days of the symptoms, which were then referred to as “apotemnophilia” or BIID, it was said that there were predominantly male homosexuals. This number has decreased significantly, more and more women and heterosexual fathers have been added; as data from the study by Diana Becker from our group (see Figure 4) show, but still—for unknown reasons—the proportion of homosexuals and bisexuals is nevertheless significantly higher than in the general population [11].

**Figure 5:**
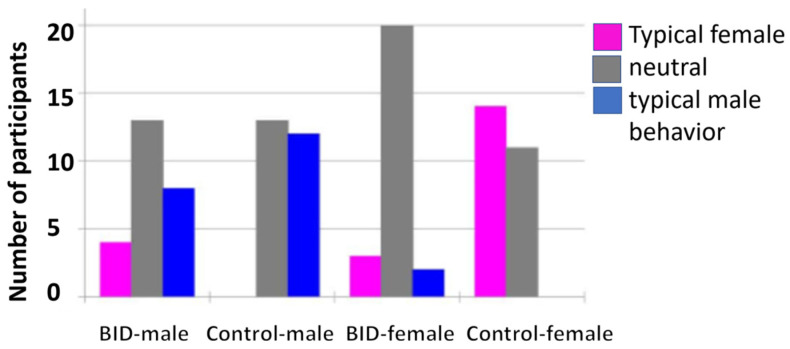
Results of Charleen Scupin’s study on male behavior in women and female behavior in men [12].

People who suffer from trans identity face similar problems. They feel uncomfortable in a female or male body that does not match their mental sense of gender. Similar to BID, this feeling exists from early childhood. So far, there has been little explanation for the extremely large number of transgender people in the BID group, i.e., in short, people who need gender reassignment surgery. Even today, most people still feel that they belong to their gender and show typically male or typically female ways of thinking and behaving. Charleen Scupin [12] from our group explored the question of whether this sense of gender belonging is as fixed in the BID group as it is in a matched average population group? She found that the BID men showed more typical female behavior than the control group; the effect was not as strong in women, but even here, more women showed typical male behavior than in the control group (see Figure 5). It is possible that BID is a general problem of identification with one’s own body.

Around 10 years ago, Antiona Ostgathe [13] addressed the question of whether and what parallels there are between BID and trans identity (transgender, transsexuality). She found a wealth of similarities between the two groups, notably: existence of body parts that are not felt to belong to one’s own; perception of a feeling that the biologically assigned body does not correspond to the mental body image; mimicking the desired appearance for approximation and testing long before surgery; no logically justifiable explanation can be given for the desire to change one’s body; feelings of guilt and shame at some stages of development; gathering material related to interest and alignment with experienced identity and shame for gathering that material [13].

**Figure 6:**
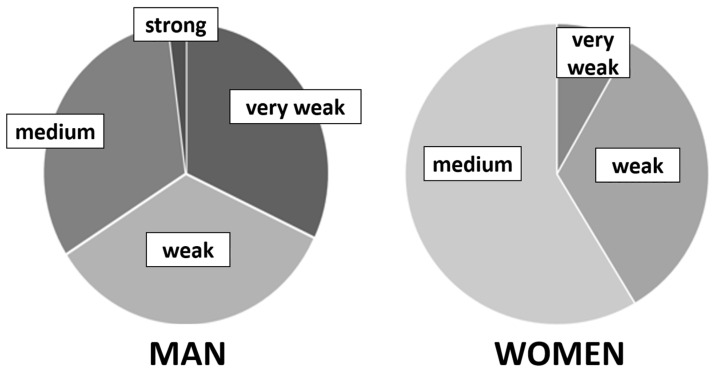
Distribution of the erotic component in BID for 50 men and 12 women in Elmas Özelik’s work [15].

There are also overlaps between BID and a certain paraphilia, which is usually referred to as “mancophilia” (love of deficiency), but also referred to as, with an ugly term, “deformation fetishism”. Most of those affected are men, and in particular the sight of amputation stumps leads to sexual arousal. Countless photos are offered in appropriate forums on the Internet, e.g., for masturbation. Many sufferers try to get to know a partner who has an amputation. In 2013, Lea Pregartbauer [14] from our lab asked people affected by BID about this erotic component. After all, around 61% admitted that amputations also contain a strong sexual component, 17% admitted a moderate participation, but on the other hand, 22% said that they felt no erotic interest in amputations. However, their work included only 18 BID participants. In 2020, Elmas Özelik [15] repeated this question in her work, but came up with significantly weaker numbers. Around a third of the participants had a very weak, weak or medium sexual component, and hardly anyone had a strong erotic interest. Women predominantly had medium or weak erotic involvement (see Figure 6).

In her study, which has already been mentioned above, Maria Garbos [3] tried to determine whether BID sufferers (in particular, with a need for amputation) might have a stronger sexual component than sufferers with a need for other disabilities, but she found no differences between the three groups: (1) amputation, (2) paralysis, (3) others.

### 2.3. Motives

Interestingly, as transgender people, those affected by BID are just as unable to answer the question “why?” The answers are rather vague, i.e.: “*I really don’t know. I simply want it. I feel where the stump would end up in my thighs and feel a strong ‘desire’ (…) to live with two thighs blunt”.*

*“I just feel like my left leg is ‘too much’ that it shouldn’t be there, just a stump instead. (…) It does not belong to the true image of my body. But I don’t find it repulsive and I don’t hate it either”.*

*“The leg/finger does not seem to belong to the body. They’re there, but they wouldn’t be missing even if they were gone. The amputation would complete the body. Only then would it be how it feels/should be”.*

### 2.4. Delusion and BID

The most naïve, succinct popular explanation is that someone wishing to have an amputation or paralysis must be “*crazy*” or “*insane*”. Both are wrong. Delusional patients are usually schizophrenic, and it is quite possible for such a psychotic to mutilate his hand in a delusion, e.g., because it tempts him or her to sin. However, schizophrenics only feel the urge to have an amputation during an acute episode; outside of the push, they almost always regret the assault. In 2020, Lebelo and Grobler [16] described a 38-year-old, unemployed, single male with no children and with an elementary level of education. The authors wrote: “He had a 4-year history characterised by ongoing persecutory delusions, as well as auditory hallucinations. He was brought to the Emergency Department by ambulance because he was found to be bleeding profusely from his scrotum in the toilet of a petrol filling station. He alleged that he had cut open his scrotum to remove his testicles before his ‘tormentors’ could do so. He stated clearly that he did not want to die because he valued his life. This was therefore not an attempt at suicide. He was initially admitted to the urology ward and then referred to psychiatry”. The multidisciplinary team diagnosed him with schizophrenia and treated him with neuroleptic medication. He responded well to haloperidol 2.5 mg orally in the morning and 5 mg orally at night.

In contrast to this case, in BID sufferers, the need is lifelong, with varying degrees of intensity, but permanently and chronically, and they obviously do not regret the amputation, which we will discuss at the end of this review.

Attempts to find typical personality profiles in BID sufferers have so far led to nothing. In our group, we have used the following psychological tests, for example, in various works: Giessen Test, Trier Integrated Personality Inventory, Symptom Check List, Rosenzweig Picture Frustration Test, Stress Management Questionnaire, Freiburg Complaint List and various others. On average, a consistent deviation in one personality domain was not found. Mia Langbehn [17] from our lab tested conflict management strategies in 47 BID sufferers, and she found an increased value for the “turn against one’s own ego” scale, but it was still in the average range, and it remains to be researched whether this value really has importance. The fact that those affected by BID are not delusional shows, among other things, that they are absolutely aware of the disadvantages of the desired disability. A delusional schizophrenic cannot be talked out of his delusions of persecution, illness, jealousy or megalomania with logical arguments. On the other hand, those affected by BID weigh this carefully. So, when asked about the pros and cons, someone stated the following:

*“PRO: I would finally be myself; would feel whole; would be inwardly liberated; secondary: my courage would give me momentum and more courage.*

*CONTRAS: I would be disabled: physically (slower, more effort required for many activities, possibly phantom pains or disturbing sensations, residual limb pain, faster wear and tear of joints in shoulders, arms and hands, back problems, need for aids, …); mentally (risk that I’ll regret it, that I’ll be ashamed, that I’ll somehow suffer from it, …); social (no longer belonging, outsider, always the smallest, shocking sight for some people, suffering of my relatives, especially my parents, for many hardly attractive anymore sexually, …); economical (what would my customers say? Could I continue to do everything in my job like this? More expensive standard of living, e.g., transport, travel, …); surgical risk; Time required for surgery, healing, rehabilitation, procurement of aids (and that again and again). In examples: I like to travel by train and also at very short distances–in a wheelchair on the train, that’s maybe easy for 20% of the trains and stations, otherwise it takes a lot of planning. In a full bar where everyone is standing, do I want to be the only one sitting in a wheelchair, at bum height? (…)”*

### 2.5. Events in Childhood

Since Sigmund Freud, many psychologists have been keen to look for all the causes of mental illnesses in childhood, and the mother is almost always the real culprit. Catharina Obernolte [18] from our group published a first paper on this question in 2015. She had essentially focused on overprotection or child neglect, but in the end the only significant result of her work was that the BID participants were simply able to remember their first encounter with a disabled person much better than the control group. In fact, most ordinary adults can only vaguely remember when they were in contact with an amputee or a wheelchair user as a child. This memory was burned crystal clear and alive in the brain of BID sufferers. For example, those affected reported:

*“There was a boy who had one leg in a black metal splint with leather straps and he had a pretty bad limp. I thought it was great”.*

*“When I was about 10, my hairdresser told me that his colleague (…) had lost a leg in a motorcycle accident–I was electrified and weeks later I was still drawing guys with only one leg”.*

*“When I was growing up (…) I played Robin Hood with friends, and because I saw on TV that one of his troops had his hand cut off unfairly by the sheriff for stealing, I put a sock over it and played it. I’d really like to know if the others noticed what that meant to me”.*

*“In 1974 I was eight years old. At some point on a summer afternoon my eyes fell on a young man in a wheelchair. To this day, I still feel drawn to him in an inexplicable way. His thin legs, his straight back, whose upper part seemed to balance itself on the lower part with every movement, and his strong upper arms were burned indelibly into my memory”.*

### 2.6. Gain from Illness and the Desire to Be Cared for

One of our most exciting studies in recent years was that of Katja Gutschke [19] from our lab (2017). She had managed to interview five people who wished to be blind, some of whom had even successfully implemented this wish. The respondents all reported that they were overwhelmed by normal vision and could only find inner peace with their eyes closed. For these people, being blind meant relief from seeing that they felt as tiring. The question that automatically arises here is whether this also applies to other types of disabilities. In fact, many BID wheelchair users report an incredible sense of calm as soon as they sit in the wheelchair. Suddenly, you are more relaxed, more balanced and more productive, they say.

Here, in 2015 Annika Kroboth [20] had asked about primary and secondary gains from illness, i.e., whether it is important for people affected by BID to be cared for, whether they believe that people with disabilities are treated considerately, that they get more sympathy and that they are relieved of obligations. The approach was highly interesting, but Ms. Kroboth found no significant differences between the BID and control groups. Thus, gaining from illness does not seem to be really decisive for the need for disability. On the contrary, the data from their study suggest that the exact opposite is the case. Many of the BID group want to prove that they can master their lives independently, even with a prosthesis or in a wheelchair. They see the disability as a challenge. For example, when asked about their motivation, some said:

*“Taking the challenge and passing it”.*

*“A boundless curiosity to know what it’s like to be an amputee”.*

*“The knowledge that I’ll do it with one foot”.*

Oscar Pistorius sprints faster than able-bodied runners; at the 2008 Olympics in Beijing, he was not allowed to start because of his prostheses: they would prefer him. *“Just because I don’t have legs doesn’t mean I’m disabled”,* said Oscar Pistorius. In fact, not only at the Paralympics, but also, for example, in cinema films, there is a certain fascination with the portrayal of disabled people; a leg amputation is a must in every pirate movie; classic is, e.g., the film “Planet Terror”, in which a woman has a machine gun instead of a prosthetic leg, with which she shoots zombies. TV series from the 1970s and 1980s such as “The Six Million Dollar Man” or “The Bionic Woman” portray people who actually only consist of spare parts, but precisely because of this they have powers that “undamaged” people are not able to achieve. One idea was that BID sufferers watched such series in childhood and then overidentified with the series heroes portrayed. To our disappointment, hardly anyone knew these films.

### 2.7. The Aesthetics of Asymmetry

**Figure 7:**
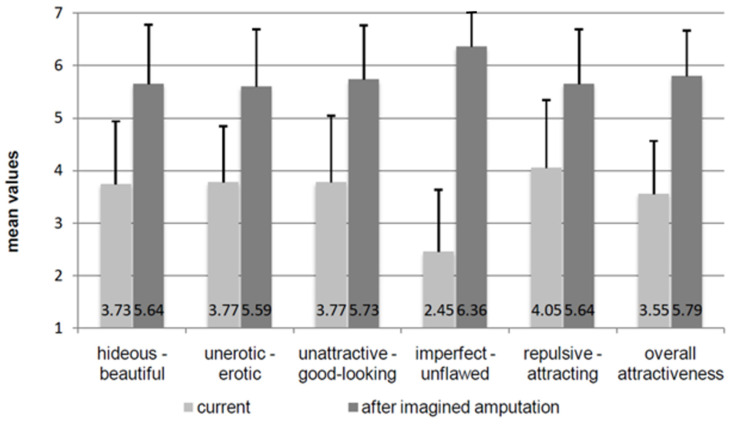
Assessment of one’s own attractiveness and erotic charisma in the intact and in the disabled body from the data of the study by Lisa-Lucie Aner.

In conversations with those affected, it became obvious that amputees were simply considered as more beautiful and attractive than people on two legs. In 2018 Lisa-Lucie Aner [21] from our group showed BID-affected persons pictures of people in the complete state and with missing arms and legs. In fact, those affected by BID rated the pictures with amputees as more attractive and beautiful. In the second part of her work, the participants were asked to rate themselves in their current (non-amputee) condition on bipolar scales (e.g., not attractive–good looking) and then give the same rating again for the desired disability. Figure 7 shows very clearly that the appearance after the loss of the body part is rated as significantly more attractive and erotic [21].

### 2.8. Pain and BID

**Figure 8:**
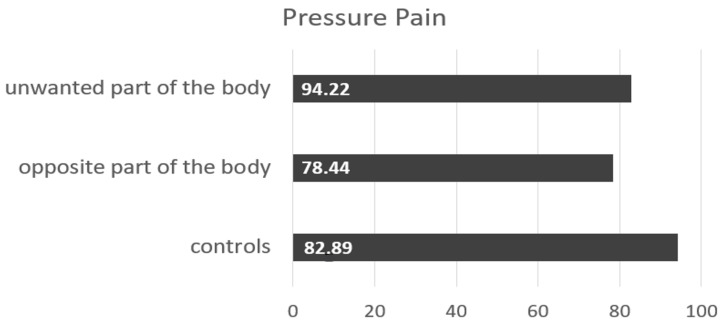
Feeling of pain due to pressure in the study by Maryam Tabesh [22] in the body part with need for amputation, in the opposite part and in controls not suffering from BID.

The research of Maryam Tabesh in 2023 deals with the pain perception in patients with body integrity dysphoria [22]. In her primary interest was the question of whether the pain perception differed between the wanted and unwanted limb. A sample of 10 participants were divided into two groups: BID sufferers (n = 5) and a matched control group (n = 5). Three different measuring instruments were taken to investigate the pain threshold. The pressure gauge FDN 200 was used for the pressure pain; the TSA2 Medoc was used for cold and heat pain and for touch sensitivity, Opti-Hair2 filaments were applied. The results of her investigation revealed significant correlations between heat pain threshold comparing the wanted and unwanted limb of the BID patients (*p* = 0.036), as well as the comparison between BID patients and the control group (*p* = 0.041, see Figure 8). 

### 2.9. Neurological Causes

Necessarily, the feeling for our body is anchored in the brain. In the somatosensory cortex, there are areas in which one perceives the own body. In the temporoparietal area, there are areas that are more complex and tell us what is and is not part of the body. Drugs such as LSD, but also medications such as ketamine, can change the way users feel about their body, and one can even become disembodied. It is therefore obvious that BID must also have a neurological basis. These connections are formed in early childhood when nerve cells connect to each other. Little babies kick to get a better feel for their bodies. It is conceivable that faulty connections form here, which is compatible with the fact that the first BID symptoms are noticed in early childhood. There is no evidence for this. It would require a long-term study of about 20 years to observe which movement patterns and which limitations small children show, who later get BID symptoms. 

Most experts today assume that BID has a brain organic cause. So far, it is unknown whether it has a genetic basis in DNA. One suspects faulty connections that may have been created before birth and, as a result, the corresponding body part is not correctly represented in the mental body image. BID-affected people can move their leg or arm, but it is not really a part of what they perceive as their own body. Here, there are counterparts from neurology, in particular, the neglect symptoms are partly similar to what BID sufferers report. As Oliver Sacks [23] wrote in the neuropsychological bestseller “The Man Who Mistook his Wife for a Hat”: “*When I asked him what happened during the night, he said frankly that he kept getting a dead, cold, hairy leg in his bed, when he wakes up at night. He could not explain where the dead leg came from and would therefore try to push it out of bed with his good arm. But it would somehow stick to his body, he couldn’t get rid of it. Every time he managed to get his leg out of bed, he would fall after it. According to him, it would be a bad joke by the hospital staff, who would put an amputated human leg on his bed night after night*”. Another neurological disorder is the “alien limb syndrome”, in which body parts are not just seen only as alien, but even make movements that their owner had not even initiated, to the extent that the hand beats their owner. Comments from BID sufferers are partly similar, e.g.: “*I have a different physical feeling about this leg than about my body and all other parts of my body. I would never give up anything more than my left leg. It doesn’t belong to my body. As previously stated, this part of the leg feels like it belongs to a stranger. Like touching a stranger’s leg. Even the feeling in my left leg is a different feeling than my right leg”.*

The scientific breakthrough was certainly the study by Gianluca Saetta and co-authors [24] from Switzerland, in which it was demonstrated that some areas in the brain of BID sufferers are wired more poorly and others better. However, it is a chicken-and-egg question as to whether the altered brain connections are a cause or a consequence of BID. Both are conceivable.

Nevertheless, there are objections to the theory of a neurological cause. Above all is the fact that the need for a disability can change. It almost never changes to completely different body parts, e.g., from leg to arm, but it does change from one leg to the other, which is mostly for rational reasons, often because the target leg is healthy, but the leg you actually wanted to keep developed, e.g., an arthrosis, and it just seemed more reasonable to the BID-affected person to have the bad leg amputated. Another unsolved question is why BID focuses on limbs and senses. We know nobody who feels the need to get rid of the nose, ears, lips or other parts of the body.

### 2.10. Therapy

**Figure 9:**
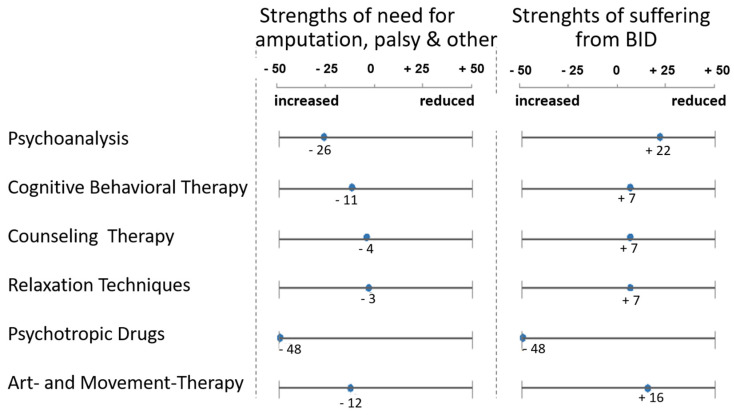
Results of the study by Katharina Kröger [25] on the usefulness of different forms of psychotherapy and pharmacological treatment.

Unless we find the cause for BID, it is difficult to propose a therapy. Still, the question that is perhaps most helpful for all those affected would be whether and how one can get rid of the suffering caused by BID. Some of them say they would very much like to keep their leg and get rid of the BID pressure; for others, it is part of their personality. In 2014 Katharina Kröger [25] asked the participants in her study about their experiences with different types of psychotherapy, including pharmacological treatment. Most of the procedures (behavioural therapy, talk therapy, relaxation techniques) were of practically no use; deep psychological or psychoanalytic treatment at least reduced the psychological strain caused by the urge for amputation, paralysis, etc. Psychopharmacological medication was unanimously assessed as negative. Body-oriented forms of treatment were judged quite differently, but many, who had tried them, noticed that the psychological strain caused by the mental focusing on their own body increased rather than decreased (see Figure 9).

Ultimately, these results suggest that BID does not have a purely psychological causation, but primarily a neural basis (although one does not exclude the other). Psychotherapy also has little effect on transgender people; the feeling of mentally belonging to the opposite sex is so deeply rooted that one cannot expect major changes here with the usual psychotherapeutic methods. This also seems to apply to BID. Of course, both groups also benefit from psychotherapy, because they can learn to cope better with the discrepancy between the mental and biological body. Healing through psychotherapy alone is not to be expected, but the behaviour in dealing with BID seems to increase the desire to fulfil the need. Imitating the disability obviously has positive effects, and those affected feel comfortable doing so. This works in the sense of positive reinforcement, which means that the need to actually acquire the disability becomes stronger and stronger, until those affected actually do it. However, as with many wishes, the feeling is no longer so euphoric when you have achieved your goal.

If all therapy is of little or no use, does amputation or permanent use of a wheelchair have a beneficial effect? In 2012, we conducted an initial study with Sarah Noll from the University of Hildesheim in cooperation with Peter Brugger, in which we interviewed 19 “successful wannabes” [5]. It was interesting that around half of them stated that the amputation had been performed by a doctor. Three people in the study were looking for paralysis and had achieved the desired condition largely through prolonged wheelchair use and leg muscle atrophy. None of the 19 respondents said they regretted their decision. Of course, all reported problems in daily life, such as in the following statement:

*“Everything takes more time and is more tiring. I can’t do everything I’ve done before, especially (at least for now) hiking, biking, volleyball and so on. Also, working in the garden is not as easy as it used to be, e.g., mowing the lawn on a hill is impossible for me. My wife has to do all these things now. I have to learn to walk with a prosthesis, it was difficult and tedious. Walking outdoors (currently) still requires absolute concentration and attention, especially when the ground requires it and I have problems recognizing the surroundings as well as I used to. Climbing stairs is annoying and my radius of action is limited. I needed a lot of time for physical therapy and walking school. But all of these cons outweigh the pros, which means I see them as achieving the big goal”.*

**Figure 10:**
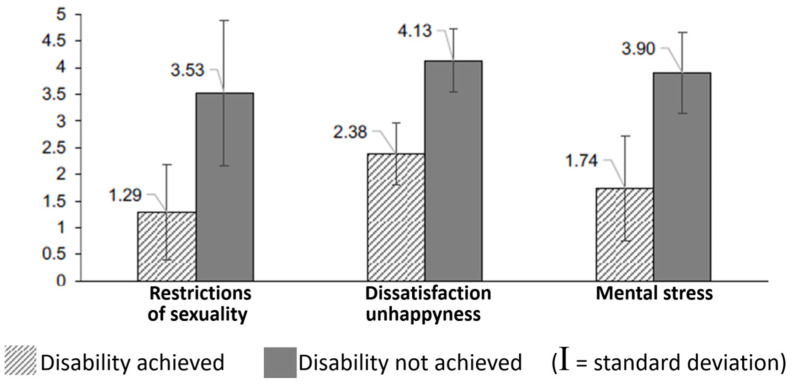
Comparison of a group of “successful” wannabes with a larger group that had not yet reached the target state in Buket Saricicek’s work [6].

Overall, the data from Sarah Noll’s work showed that people who achieved their desired state were happier, healthier and more balanced in all areas of life. (see Table 2). Another study on this topic was carried out by Buket Saricicek in 2021 [6]. She compared 21 sufferers who had achieved their desired state with 98 sufferers who had not (yet) achieved it. Figure 10. shows that the impairments in sex life, general dissatisfaction and psychological stress are significantly lower in the group of the “successful” than in the people who have not yet reached their goal. Still, until now we only have little evidence to prove that amputation really helps to cure this condition. Another problem is that it is very difficult to convince doctors and insurance companies to facilitate legal amputation. Even if they agree, patients may go through horrible consequences if the social environment finds out that the amputation is intentional, i.e., not caused by accident or illness.

## 3. Limitations

The problem with research on Body Integrity Dysphoria is that most affected individuals are embarrassed to have it and are reluctant to participate in scientific studies for fear that their true identity will be revealed. This required considerable persuasion. As a result, the number of participants is small, and it is often the same people who took part in the studies. Another limitation is that, in these studies, we mainly conducted online surveys with test methods we developed ourselves. This means that data can easily be falsified, since the person is not asked directly, and the information has to be relied upon. However, it has turned out in the past that those affected by BID are very interested in finding out what they are actually suffering from, why and what can be done about it. We therefore assume that the information given is largely true. Online surveys are also widespread today and are carried out in countless studies. In addition, a large proportion of the participants in our studies have also become personally known over the years. Those affected by BID could be encouraged to found an association, and they meet regularly.

## 4. Conclusions and Recommendations

In 2004, that is, about 20 years ago, the author of this article started studying this strange phenomenon. While researching body modification, he came across people who wished to have an amputation; at that time, this phenomenon was called “apotemnophilia”. At the time, it was completely unclear whether BID sufferers were mentally ill, whether they were schizophrenic, for example, or whether it was self-harm in borderline patients? The first studies then quickly revealed that those affected by BID are not more mentally ill than the rest of the population. The assumptions from Michael First’s study [2] were also largely confirmed in our data. The early onset is typical; even as children, those affected often have the feeling that they are in the wrong body. It may be a genetic component, but it is more likely that the brain was “wired” incorrectly before birth and a part of the body was not in the awareness of what belongs to the body—and what does not. The mental feeling is that of a disabled person, but the outer body is healthy. In other studies, we found parallels to transgender people who have similar problems. Both involve a disruption in the identity between the outer and inner body. A surprising number of people affected by BID also have no fixed gender role, and it is not uncommon for them to suffer from both (BID and transgender). There is certainly a common ground here. The sexual component and overlap with mancophilia (i.e., deformation fetishism) is little researched. We are now convinced that BID has a neurological cause, but over the course of life, psychological components are added. Pretending behavior, i.e., trying out the disability, is perceived as pleasant; those affected say that they feel positive in the wheelchair or with crutches. The biggest unsolved problem is what actually falls under BID. Our scientific studies started with the need for an amputation, then came people who wanted paralysis (up to paraplegia). In one of our studies, we also found parallels to the urge to be blind. Yet, what about people who want to be deaf, mute or incontinent? Overall, the data underline that—at least so far—amputation (or other ways of achieving the desired body condition such as atrophy of the muscles) is the only way to really eliminate the suffering caused by Body Integrity Dysphoria and to help those affected live a normal life—whatever “normal life” means.

## Figures and Tables

**Table 1:** Data from Maria Garbos’ study from our group on the frequency of occurrence of different types of disability needs.

Type of Disability	Percent (n = 213)
Amputation	47.7%
Palsy	23.5%
Amputation + palsy	5%
Palsy + incontinency	4%
Blindness	2%
Amputation + others (e.g., stutter)	1%
Amputation + palsy + incontinency	1%
Palsy + incontinency + deafness	1%
Amputation + edentulous	1%
Amputation + blindness	0.5%
Palsy + incontinency + blindness	0.5%
Edentulous + dumbness	0.6%
other	2.5%

**Table 2:** Results of Sarah Noll’s study [5] in comparison before reaching the desired physical condition and afterwards on a scale from bad = −50 to good = +50.

	Pre (M ± SD)	Post (M ± SD)	Wilcoxon-Test
General satisfaction with life	−22.8 ± 20.5	44.7 ± 7.7	*p* < 0.01
Job satisfaction	23.2 ± 28.9	43.7 ± 7.6	*p* < 0.01
Private satisfaction	2.6 ± 26.3	43.2 ± 7.6	*p* < 0.01
Health status	24.7 ± 29.5	42.6 ± 12.6	*p* < 0.05
Sexual satisfaction	16.3 ± 26.3	38.4 ± 21.2	*p* < 0.01
Body identity	−19.0 ± 29.4	47.4 ± 5.6	*p* < 0.01

## Data Availability

The original data are only partially available. Most exact data can be found in the original publications.
